# Gender differences in representation, citations, and h-index: An empirical examination of the field of communication across the ten most productive countries

**DOI:** 10.1371/journal.pone.0312731

**Published:** 2024-11-20

**Authors:** Manuel Goyanes, Esperanza Herrero, Luis de-Marcos

**Affiliations:** 1 Department of Communication, Carlos III University of Madrid, Getafe, Spain; 2 Department of Communication, University of Murcia, Murcia, Spain; 3 Department of Computer Science, Universidad de Alcalá, Alcalá de Henares, Spain; Lamar University, UNITED STATES OF AMERICA

## Abstract

Women researchers have been shown to be underrepresented in science, especially among the most productive scholars. This is especially relevant in the social sciences and humanities fields, where gender parity is closer, but disparities among top scholars are still pronounced. The gender gap in the field of communication has been explored from several approaches, but studies focusing on gender differences in representation, citations, and h-index are rather scarce. Drawing upon data retrieved from SciVal, we conducted a comparative study of the top 500 and top 100 most productive scholars (N = 5000) for each of the ten most productive countries in communication research in the 2019–2022 period: the United States, the United Kingdom, China, Spain, Germany, India, Australia, Canada, Italy, and the Netherlands. The results indicate a consistent underrepresentation of women, particularly among the top 500, across countries. Despite women being cited more frequently than men in some countries over shorter time frames, a gender bias persists favoring men, particularly when considering the h-index. All in all, our study shows that, despite hints of gender equality in citation patterns, the gender gap still constitutes a structural part of the field of communication when addressing gender representation in research productivity and long-term dynamics of research impact.

## Introduction

Gender inequalities in science are a global phenomenon, stable across different fields, countries, and regions, and with strong historical and social roots [1, 2]. Among other approaches, this imbalance is studied through scientometric indicators that quantify the differences in productivity, representation, and citations between female and male scholars [3]. So far, most prior studies around this issue have focused on fields other than communication, finding a stable gender-gap across disciplines regarding representation and/or citations [4, 5].

In particular, there exists in the field of communication a growing interest in understanding the gender-gap in research output and citations. In fact, prior literature has found significant inequalities regarding citation patterns and practices in our field [6–9]. Our article is the first to our knowledge, to investigate gender differences in representation and cumulative impact in communication research, examining not only citation disparities within a specific time frame but also h-index discrepancies, thereby shedding light on cumulative differences.

This is especially relevant considering that most inequalities in science are the result of cumulative mechanisms [10]; thus, only noticeable in the long term. In fact, analyzing only gender differences in citations can give us a biased, stationary picture of the situation of female scholars. When more accurate, cumulative measures of scientific impact are considered, we may challenge this biased perspective and gain a better understanding of long-term dynamics structuring research impact. In our case, these are relevant because, while female scholars are more cited than men in some countries, men still appear to be favored when the h-index is considered.

To reproduce a relevant and diverse picture of gender differences in the field on a global scale, we focused on the ten most productive countries in communication research in terms of research output (i.e., productivity) in the 2019–2022 period: the United States, the United Kingdom, China, Spain, Germany, India, Australia, Canada, Italy, and the Netherlands. As major science producers, these countries do not just concentrate most of the literature produced but also have a greater influence upon research performance, trends, and thematic dynamics in communication research on an international level.

Specifically, to address the gender gap in representation and cumulative/stationary impact, we conducted a comparative study between the top 500 and top 100 most productive scholars in each country. All in all, through this work, we aim to further the research on gender disparities in the field of communication, analyzing the gender differences in representation and the impact of the most productive scholars across the most productive countries globally in the field of communication in the 2019–2022 period.

Our main findings first indicate a consistent underrepresentation of women among the most productive scholars, particularly among the top 500, across countries. Additionally, the results show that gender disparities are greater when considering long-term indicators. Women are sometimes more cited than men in a shorter time frame (e.g., 2019–2022), with female scholars having a greater median citation count than males in many countries. However, cumulative gender differences flourish when considering longer indicators, such as the h-index. In particular, our results show that gender differences in h-index are statistically significant, with women having a lower h-index in almost every country under analysis. Accordingly, gender differences in research impact appear to decrease over short periods of time; however, these differences become more pronounced when considering the cumulative impact over the years.

## Women in science

Research on the gender gap in science has found that differences between male and female scholars are stable across fields, regions, and countries, existing both in STEM and in the social sciences and humanities fields [5]. The underrepresentation of female scholars is a historical phenomenon with epistemic roots [11] that can be explained through many different factors, like the structural persistence of traditional sociocultural gender roles and stereotypes [12–14]. Gendered social expectations are often incompatible with career expectations, thus forcing female scholars into a conflicted position [15, 16] and complicating their success in a highly demanding system like academia.

Gender imbalances have also been studied from the perspective of specific gender discrimination dynamics in academia that undermine female careers. These include scientific prejudices and stereotypes [17], structural barriers [16], undervaluation and biases [7, 18], the Matilda effect and a lack of symbolic authority [6, 19], lack of female networks [20], lack of role models [21], and so on. As a result, a gender gap has formed in which women have been found to be “significantly undercited” [22: 1161], less able to succeed in a long-term academic career than men [23], underfunded and/or underpaid [24], underrecognized as referents [25], undervalued as collaborators [7], or less likely to be promoted in their academic career [26].

The field of communication research is no stranger to these dynamics. In our field, women are not only less cited than men [6–8] but also largely affected by existing citation practices and coauthorship networks [27]. Female academics in communication research are also less regarded as referents, being less included in communication degrees’ syllabi, where they have been found to roughly represent 20% of the authors mentioned [28]. From a historiographical perspective, women have also been erased from the coauthorship of foundational books of the field [29] and of most historical reviews [30, 31], and their contributions have been—and continue to be—“generally excluded” [32: 7] from our shared canon. In general, in our field, women have had more problems than men when constructing networks and finding mentors; they have had problems balancing career and gender expectations; and they have faced sexual harassment and other forms of gendered de-legitimation [33].

According to McCusker [34], it is not the individual impact of these gendered practices and stereotypes, but rather the cumulative harm of them all that ultimately damages and undermines women’s careers. Thus, gender differences must be addressed from a holistic perspective, as the result of a complex, long-term, structural epistemic injustice.

## Gender differences in representation and impact

Success in contemporary science is intrinsically linked to high levels of productivity, impact, and quality [35]. All of these indicators combined are the materialization of science capital [36], a cumulative form of authority that is essential for success in a “credibility economy” like academia [17].

The widespread use of bibliometric indices (number of publications or citations, h-index, etc.) measures science success in terms of knowledge production and its impact. In particular, citations have been the measure of science’s impact, quality, and influence since the mid-20^th^ century [37]. Other, more complex, indicators are gaining popularity: The h-index combines publication and citation, assuming that “the number of citations received by a scientist is a better indicator of the relevance of his or her work than the number of papers he or she publishes or the journals where they are published,” and it is used to measure the impact of a researcher on the development of a specific field [38: 161]. The h-index has been criticized as being a potentially biased indicator towards scholars with longer careers or from less-cited fields. Similarly, the h-index does not distinguish between corresponding authors and privileges some scholars over others [39], perpetuating Matthew effects. Consequently, academic careers are stratified, ranked, and hierarchized according to some or all of these indicators of research performance and success [37, 40].

Despite the growing percentage of women researchers in almost all disciplines, prior literature finds consistent gender differences in representation when focusing on academic success [4]. In particular, most humanities and social sciences fields are now “close to parity” [18: 75], including the field of communication research. Despite this tendency towards equality, the gender gap is still evident among high-performing, “elite,” top scholars [41], where less female names are present. This is linked to the lack of symbolic authority women have in knowledge production, a tendency that is ultimately exemplified in the Matilda effect theory: Women are systematically less cited, less awarded, and less recognized as authorities than men [19]. All in all, the Matilda effect is the materialization of long-standing, historical, and epistemic conceptions about gender that link femininity to the irrational and maleness to the rational [11]. In general, in most epistemic traditions, women have been considered to represent emotion rather than reason [11], and thus have been either relegated to a secondary position in academia, or excluded from knowledge altogether [42]. Even today, women systematically have more trouble being considered an authority in scientific and knowledge matters [17], and gender still plays a major role in the symbolic fight for science capital [36]. This is tremendously damaging considering that academia constitutes a “credibility economy” [17], where success is dependent on being listened to by others. Epistemic prejudices against women result in what Fricker [17: 1] addresses as “testimonial injustice”: Hearers systematically give a “deflated level of credibility” to female scientists, drawing on stereotyped conceptions about gender and knowledge. Ultimately, this form of injustice has undermined and continues to make difficult women’s careers in academia.

In general, women have been found to be less likely to “climb the academic ladder” [40]. The “leaky pipeline” theory [43, 44] addresses this phenomenon by explaining that the more demanding of high-performance the academic career gets, the more female talent is lost. As a result, despite the higher presence of women among the lower steps of academia—especially as PhD students and other low-productive figures [45]—there is a significant loss of female talent throughout the late stages of the academic career. As a consequence, women are significantly underrepresented among the top scholars in most disciplines [46].

Ultimately, this loss undermines plurality and diversity in science, conforming to narrow and unidimensional fields that look at the world from only one point-of-view. Going back to Fricker [17], this constitutes a form of “hermeneutical injustice.” Hermeneutical injustice can be explained as the result of testimonial injustice: When the voices of some members of epistemic communities are systematically and deliberatively not heard, the knowledge produced is incomplete, and a gap in collective interpretive resources is created [17: 1]. This gap is ultimately a call to pluralize the voices in science, in the conviction that, the more plural epistemic communities are, the more capable of addressing and knowing reality they will be. When we lose diversity in academic spaces, we are losing a chance to produce valid, trustworthy, plural, and ethical knowledge about the world.

In communication research, women earn more PhDs than men [30, 45], but are still a minority among full professorship and high-ranking positions [47]. Although prior research has studied the role of women in the field of communication, gender differences in representation remain largely unexplored in our field. Very few studies have analyzed the gender imbalance in communication research from a scientometric perspective, with most of them addressing citation patterns and practices [6–8, 27], but with a lack of reference to the consequences of these gendered practices for the configuration of our field. In this paper, to fill the gap in the literature, we analyze gender differences in representation and impact in the field of communication research.

## Hypotheses and research questions

Despite the approximately equal proportion of male and female scholars in the social sciences and humanities fields [18], female talent is lost throughout the stages of the academic career, with women being overrepresented in the early-career steps and low-productivity roles, like those of PhD students and underrepresented in senior positions and high-productivity roles [47]. This phenomenon is referred to as the “leaky pipeline” [44]. Considering the above, we present one research question and three hypotheses as follows:

RQ1) Assuming the gender proportions in the pooled sample (N = 500, M_male_ = 57.20%, M_female_ = 42.79%; N = 100, M_male_ = 59.1%, M_female_ = 40.9%), are there statistically significant deviations from this representation in each country under analysis considering: 1a) the top 500 scholars, and 1b) the 100 top scholars?

H1) Assuming equal gender proportions (male/female 50%) between 1a) the 500 and 1b) the 100 most productive scholars in the most productive countries in the field of communication, there are statistically significant fewer female scholars in each country under analysis and in the pooled sample; and
H2) Female scholars a) have statistically significant less citations, and b) have a lower h-index, than male scholars among ab_1_) the top 500 and ab_2_) the top 100 scholars for each country under examination and the pooled sample.

Considering, as stated before, that women have also been found to be systematically less cited than men, we present the third hypothesis:

H3) Controlling for research productivity, female scholars a) are statistically significant less cited, and b) have a lower h-index, than male scholars among ab_1_) the top 500 and ab_2_) the top 100 scholars for each country under examination and the pooled sample.

## Procedure

To answer the research question and test the hypotheses, data was directly gathered from SciVal, a platform that provides scientometric data of research impact and performance with data computed from Scopus. Specifically, data was directly downloaded from the platform on the 4^th^ of May, 2023, with the 26th of April being the last date the data was updated, according to the platform. For analytical purposes and in order to provide empirical findings of gender differences of research impact from the countries that produce a large percentage of the world research output in the field of communication, the ten most productive countries in terms of paper production were selected for further analysis. This data was also computed by using data directly from SciVal.

Accordingly, the sample included data from the following ten most productive countries in the world in the field of communication during 2019–2022: the United States, the United Kingdom, China, Spain, Germany, India, Australia, Canada, Italy and the Netherlands. By default, SciVal reports scientometric data of the 500 most productive authors for each country, and this is the data that was used to form the sample for each country. However, since productivity and citation measures range significantly from the top one to the top 500 scholars, we also decided to focus on the top 100 most productive scholars for each country and run a complementary analysis to add more nuances. This means our analysis covers either 100 or 500 scholars per country. Therefore, when focusing on the top 100, we have a total of 1,000 scholars, and when focusing on the top 500, we have a total of 5,000 scholars.

The variables and measurements considered in the analysis, framed between 2019–2022, were: the name of the scholar (N_per_field_ = 500 or 100, N_total_ = 5,000 or 1,000), the research output of the scholar (i.e., number of articles published; M_5,000_ = 4.92, SD_5,000_ = 3.80; M_1,000_ = 9.31, SD_1,000_ = 5.32), the number of citations of the scholar (M_5,000_ = 32.02, SD_5,000_ = 56.89; M_1,000_ = 68.48, SD_1,000_ = 89.88), the h-index of the scholar until the date of data collection (i.e., historical data; M_5,000_ = 12.30, SD_5,000_ = 11.66; M_1,000_ = 14.95, SD_1,000_ = 12.58), and country of the scholar (i.e., the ten most productive countries reported above). Zero-order correlations for the top 100 and top 500 for the pooled sample are reported in Table 1.

**Table 1:** Zero order correlations.

	Productivity	Citations	h-index
Productivity	-	.603***	.146***
Citations	.596***	-	.236***
h-index	.198***	.276***	-

*Note*. Pearson correlation entries below the hyphen are N = 1,000, while entries above the hyphen are N = 5,000.

*** p < .001.

Namsor software was used to code the gender by using its split name functionality improved with country. It provided the gender and the probability of correct classification using data mining software. At a threshold of .625, we applied the Namsor gender for 4,707 (94.14%) scholars and manually coded the remaining 293 (5.86%). Namsor documentation suggests that if the probability of the returned gender is between .45 and .55, the name could be interpreted as a unisex name. Namsor is an accurate tool that has been consistently used in bibliometric gender studies [48].

## Data analysis

To test H1a and H1b, we ran a chi-square goodness-of-fit test. Specifically, we assumed equal gender proportions (50%), and tested deviations from this expected percentage. Since the total sample were the top 500 most productive scholars for each country, this sample size was used for testing H1a, while the selection of the top 100 were computed for testing H1b. Accordingly, RQ1 examines gender proportions of the most productive scholars within the most productive countries by considering two different samples: the top 500 and the top 100.

Similarly, once we knew the gender proportions across all countries (i.e., the pooled sample: the actual gender distribution within the dataset), the chi-square test of RQ1 assumed these values and explored gender differences in proportions among the top 500 scholars (RQ1a), and the top 100 (RQ1b), across countries to understand potential deviations from the expected proportions in each country under examination. As citations and h-index distributions were non-normally distributed (*p* < .05), a rank-based nonparametric test was used. Specifically, we implemented a series of Mann-Whitney U tests to understand (for H1) whether citations and h-index (measured on a continuous scale) differed based on gender (which had two groups for the purpose of the tests: male and female). Since distributions were similarly shaped for citations and h-index for both males and females in N = 100 and N = 500, we investigated differences in medians.

Finally, to test H2, we implemented a bootstrap OLS-regression, both for the top 500 and top 100 scholars. Since the assumption of normality of observations were not met, a bootstrap OLS-regression was implemented, as this regression procedure is highly recommendable for non-normally distributed variables. Regressions were run in 1,000 bootstrapped samples, bias-corrected and accelerated to produce confidence intervals set at 95%. Means and standard deviations across countries for productivity, citations, and h-index are reported in Table 2. To account for possible overlap between citations and the h-index in measuring scholars’ impact, we controlled for the effect of each when examining the other (i.e., we included citations as a variable in the regression when predicting the h-index, and controlled for the h-index when predicting citation levels). In addition, there are strong theoretical reasons to assume that cumulative levels of prestige (i.e., h-index), may influence scholars’ impact measured in citations in a particular time frame, and that citation scores during a particular time frame (as in our study) explain part of the variance of cumulative h-index. However, as a robustness check, we also ran all bootstrap regression analyses controlling only for research productivity, which are reported in S1–S4 Tables. Research productivity is controlled by including in the regression the number of papers published by each scholar during the time frame under study. Results are similar in the direction, significance, and strength of the associations.

**Table 2:** Productivity, citations, and h-index means across countries.

	Productivity		Citations		h-index	
	N = 500	N = 100	N = 500	N = 100	N = 500	N = 100
United States	11.00 (4.01)	17.35 (4.45)	94.26 (94.13)	170.98 (133.33)	16.14 (11.36)	18.93 (10.91)
United Kingdom	4.50 (2.38)	8.17 (3.09)	35.54 (50.98)	78.84 (76.73)	13.19 (13.05)	18.83 (17.76)
China	4.70 (2.80)	8.92 (3.89)	13.02 (28.19)	20.32 (29.53)	12.14 (11.72)	13.93 (12.40)
Spain	6.11 (3.34)	11.17 (4.32)	24.83 (31.91)	52.32 (49.92)	7.00 (6.62)	8.57 (5.31)
Germany	5.07 (2.79)	9.78 (2.61)	38.36 (58.94)	87.59 (69.02)	11.53 (11.38)	14.25 (9.34)
India	3.13 (2.11)	6.03 (3.24)	8.31 (18.72)	13.78 (25.35)	8.50 (9.17)	11.50 (13.76)
Australia	4.12 (2.55)	8.26 (2.81)	28.50 (40.04)	58.44 (56.55)	14.13 (13.10)	14.19 (9.98)
Canada	3.05 (2.60)	6.30 (4.48)	18.03 (33.35)	42.48 (61.85)	13.39 (13.66)	15.74 (14.53)
Italy	3.50 (3.82)	7.63 (7.72)	16.49 (32.72)	36.71 (63.27)	13.00 (11.09)	15.33 (12.38)
Netherlands	4.03 (3.83)	9.56 (5.69)	42.84 (77.83)	123.41 (128.39)	14.01 (10.97)	18.24 (11.89)

*Note*. Standard deviations in brackets

## Results

H1 predicted that, assuming equal gender proportions (male/female 50%), between a) the 500, and b) the 100, most productive scholars in the most productive countries in the field of communication, there are statistically significant less female scholars in each country under analysis and in the pooled sample. Results of the chi-square test (H1a) reported in Table 3, revealed that for the top 500 scholars in all countries under examination (and the pooled sample), excepting the United States, Spain, Australia, and the Netherlands, there are statistically significant more male scholars as most productive authors than females (see residuals in Table 3 to assess deviations from expected values).

**Table 3:** Gender representation among the top 500 and 100 most productive scholars in the ten most productive countries in communication assuming equal proportions.

	N = 500				N = 100			
Country	Male	Female	Residual	χ^2^(df)	Male	Female	Residual	χ^2^(df)
United States	261	238	-11.5	1.06(1)	55	45	-5	1(1)
United Kingdom	272	228	-22	3.87(1)*	57	43	-7	1.96(1)
China	307	193	-57	25.99(1)***	67	33	-17	11.56(1)***
Spain	259	241	-9	0.64(1)	58	42	-8	2.56(1)
Germany	311	189	-61	29.76(1)***	62	38	-12	5.76(1)*
India	354	145	-104.5	87.53(1)***	79	21	-29	33.64(1)***
Australia	267	233	-17	2.31(1)	53	47	-3	0.36(1)
Canada	282	218	-32	8.19(1)**	57	43	-7	1.96(1)
Italy	282	218	-32	8.19(1)**	59	41	-9	3.24(1)
Netherlands	264	236	-14	1.56(1)	44	56	6	1.44(1)
TOTAL	2,859	2,139	-360	103.72(1)***	591	409	-91	33.12(1)***

*Note*. Residual values are computed for female scholars.

* *p* < .05

** *p* < .01

*** *p* < .001

The largest residuals, and therefore the countries with the strongest gender differences in representation, were India (χ^2^(1) = 87.53, *p* < .001, Residual = -104.5), Germany (χ^2^(1) = 29.76, *p* < .001, Residual = -61), and China (χ^2^(1) = 25.99, *p* < .001, Residual = -57), while Spain (χ^2^(1) = .64, *p* > .05, Residual = -9), the United States (χ^2^(1) = 1.06, *p* > .05, Residual = -11.5), and the Netherlands (χ^2^(1) = 1.56, *p* > .05, Residual = -14) were the best-positioned in terms of gender representation among the most productive scholars. As observed in Table 3, in all countries and in the pooled sample, there are fewer female scholars than would be expected, but especially relevant are deviations in India and Germany. In total, H1a was supported in 7 out of 11 countries (including the pooled sample).

When it comes to the top 100 (H1b), results reported in Table 3 indicate that only in China, Germany, India, and the pooled sample, were there statistically significant differences in representation assuming equal gender proportions, and as in the top 500, there were also fewer female scholars. The largest deviation from expected values were registered, once again, in India (χ^2^(1) = 33.64, *p* < .001, Residual = -29), China (χ^2^(1) = 11.56, *p* < .001, Residual = -17), and Germany (χ^2^(1) = 5.76, *p* < .05, Residual = -12). The only country where expected values were higher than expected was in the Netherlands, as the percentage of female scholars among the top 100 most productive scholar was 56%. However, this difference was not statistically significant. In the rest of countries there were fewer female scholars than would be expected, but only in 4 out of 11 predictions, the difference was statistically significant (H1b).

RQ1 inquired if, assuming the gender proportions from the pooled sample (N = 500, M_male_ = 57.20%, M_female_ = 42.79%; N = 100, M_male_ = 59.1%, M_female_ = 40.9%), there are statistically significant deviations from this representation in each country under analysis considering 1a) the top 500 scholars and 1b) the top 100 scholars. Results reported in Table 4 revealed that even considering the proportions of the pooled sample (i.e., highly biased in favor of male scholars) for the top 500 scholars (RQ1a), India (χ^2^(1) = 38.49, *p* < .001, Residual = -68), and Germany (χ^2^(1) = 5.10, *p* < .05, Residual = -25), are countries where there are statistically significant fewer females than would be expected, while in Spain (χ^2^(1) = 5.95, *p* < .05, Residual = 27), the United States (χ^2^(1) = 4.88, *p* < .05, Residual = 24.4), and the Netherlands (χ^2^(1) = 3.95, *p* < .05, Residual = 22), there are statistically significant more female scholars than would be expected assuming the proportions of the pooled sample.

**Table 4:** Gender representation among the top 500 and 100 most productive scholars in the ten most productive countries in communication assuming real proportions coming from the pooled sample.

	N = 500				N = 100			
Country	Male	Female	Residual	χ^2^(df)	Male	Female	Residual	χ^2^(df)
United States	261	238	24.4	4.88(1)*	55	45	4.1	0.69(1)
United Kingdom	272	228	14	1.60(1)	57	43	2.1	0.18(1)
China	307	193	-21	3.60(1)	67	33	-7.9	2.58(1)
Spain	259	241	27	5.95(1)*	58	42	1.1	0.05(1)
Germany	311	189	-25	5.10(1)*	62	38	-2.9	0.34(1)
India	354	145	-68	38.49(1)***	79	21	-19	16.38(1)***
Australia	267	233	19	2.94(1)	53	47	6.1	1.53(1)
Canada	282	218	4	0.13(1)	57	43	2.1	0.18(1)
Italy	282	218	4	0.13(1)	59	41	0.1	0.00(1)
Netherlands	264	236	22	3.95(1)*	44	56	15.1	9.43(1)**

*Note*. Residual values are computed for female scholars.

* *p* < .05

** *p* < .01

*** *p* < .001

For the top 100 (RQ1b), in most countries males and females were similarly distributed, assuming the proportion of the pooled sample. However, India (χ^2^(1) = 16.38, *p* < .001, Residual = -19) still has statistically fewer female scholars than would be expected, while the Netherlands (χ^2^(1) = 9.43, *p* < .01, Residual = -15.1) is the only country in the sample where there are more female scholars than would be expected among the top 100 most productive scholars, assuming the proportions of the pooled sample.

H2 predicted that female scholars a) have statistically significant less citations, and b) have a lower h-index than male scholars among ab_1_) the top 500 and ab_2_) the top 100 scholars for each country under examination and the pooled sample. Results of the Mann-Whitney U tests revealed in Table 5, quite surprisingly, that for H2a_1_) in none of the ten countries under examination for the top 500, there were statistically significant differences in citations between the female and male most productive scholars, and only in the pooled sample was the difference statistically significant (*p* < .01), with female scholars (*Mdn* = 14) having a greater median than males (*Mdn* = 12). Accordingly, H2a_1_ was not empirically supported, and was even contrary to our expectations. As for H2a_2_ (i.e, the top 100 most productive scholars), there was statistically significant differences in citations between females and males in the United Kingdom and India (*p* < .05). For both countries, male (*Mdn*_UK_ = 63; *Mdn*_India_ = 8) scholars have a higher citation median than females (*Mdn*_UK_ = 42; *Mdn*_India_ = 2). Therefore, H2a_2_ was partially supported, but only in 2 of the 11 potential predictions.

**Table 5:** Gender differences in citations and h-index across countries for the top 500 and top 100 most productive scholars.

	Citations						h-index					
Country	Top 500			Top 100			Top 500			Top 100		
	*U*	*Z*	*p*	*U*	*Z*	*p*	*U*	*Z*	*p*	*U*	*Z*	*p*
United States	30,818	-0.15	.881	969	-1.86	.060	28,052	-1.87	.061	998	-1.66	.097
United Kingdom	29,211	-1.11	.264	931.50	-2.04	**.041**	24,239	-4.21	**.000**	818	-2.84	**.005**
China	28,996	-0.40	.688	1,071	-0.24	.806	26,008.50	-2.30	**.021**	1,091	-0.10	.915
Spain	30,895	-0.19	.845	1,117.50	-0.70	.483	26,221	-3.10	**.002**	1,010	-1.46	.143
Germany	28,713	-0.43	.666	1,074	-0.73	.460	22,715.50	-4.26	**.000**	750	-3.04	**.002**
India	22,932	-1.88	.060	538.50	-2.47	**.013**	19,281.50	-4.37	.**000**	761	-0.58	.561
Australia	30,265	-0.52	.602	1,238	-0.04	.961	26,515	-2.85	**.004**	1,194	-0.35	.724
Canada	29,700.50	-0.64	.517	1,202	-0.16	.870	28,021	-1.69	.090	1,058	-1.16	.243
Italy	28,178.50	-1.60	.109	1,166	-0.30	.760	23,744.50	-4.36	**.000**	854	-2.49	**.013**
Netherlands	30,112	-0.64	.519	1,095	-0.94	.343	24,805.50	-3.93	**.000**	1,044	-1.30	.191
TOTAL	2,922,328.50	-2.68	**.007**	114,553.50	-1.40	.160	2,617,455.50	-8.73	**.000**	105,421	-3.44	**.001**

*Note*. Statistically significant *p* values in bold.

As for the differences in h-index in the top 500 most productive scholars (H2b_1_), results revealed that in all countries (including the pooled sample), excepting for the United States and Canada, the differences between males and females in h-index were statistically significant, female scholars having less h-index than males. Accordingly, H2b_1_ was partially supported, with 9 out of 11 predictions confirmed. When it comes to gender differences in h-index in the top 100 (H2b_2_), results revealed statistically significant differences in the United Kingdom (*p* < .01), Germany (*p* < .01), Italy (*p* < .05), and the pooled sample (*p* < .01). In all cases, female scholars had less h-index than males. Therefore, H2b_2_ was partially supported, but only in 4 out of 11 predictions.

Finally, H3 predicted that after controlling for research productivity and h-index, female scholars a) are statistically significant less cited, and b) have a lower h-index than male scholars among ab_1_) the top 500 and ab_2_) the top 100 scholars for each country under examination and the pooled sample. Results of the bootstrapped OLS-regression in Table 6 revealed, contrary to our predictions, that H3a_1_) for the top 500 scholars, in Spain (*b =* .07, *p* < .05, ΔR^2^ = 0.5%) and in the pooled sample (*b =* .02, *p* < .05, ΔR^2^ = 0.1%), at the same level of research productivity and h-index, female scholars are statistically significant more cited than males. In the rest of countries, this association was not statistically significant. Accordingly, H3a_1_ was not empirically supported.

**Table 6:** Bootstrapped OLS regression predicting citations for the top 500 most productive scholars across the most productive countries in communication.

	(Top 500) Citations										
	United States	United Kingdom	China	Spain	Germany	India	Australia	Canada	Italy	Netherlands	TOTAL
Block 1											
Research Productivity	.52***	.52***	.15*	.59***	.44***	.10	.53***	.49***	.65***	.74***	.58***
	(1.58)	(1.62)	(0.52)	(0.95)	(1.21)	(0.76)	(0.59)	(0.49)	(1.22)	(1.05)	(0.43)
ΔR^2^	30.1%	31.5%	2.6%	37.7%	22.9%	1.4%	27.7%	25.1%	46.8%	58.4%	36.4%
Block 2											
H-index	.17***	.17***	.05	.22***	.21	.11	.14***	.07	.15**	.09**	.15***
	(0.33)	(0.16)	(0.10)	(0.27)	(0.63)	(0.14)	(0.11)	(0.09)	(0.14)	(0.21)	(0.08)
ΔR^2^	2.9%	3.1%	0.3%	4.7%	4.4%	1.2%	2.2%	0.4%	2.3%	0.7%	2.2%
Variable of Interest											
Gender_(female)_	.05	-.04	.03	.07*	.02	-.00	-.03	.03	-.00	.00	.02*
	(6.96)	(3.65)	(2.44)	(2.15)	(3.44)	(1.79)	(3.04)	(2.62)	(2.53)	(4.44)	(1.23)
ΔR^2^	0.3%	0.2%	0.1%	0.5%	0%	0%	0.1%	0.1%	0%	0%	0.1%
R^2^	33.2%	34.7%	3%	42.9%	27.4%	2.7%	30%	25.6%	49.1%	59.1%	38.7%
Adj.R^2^	32.8%	34.4%	2.4%	42.6%	26.9%	2.1%	29.5%	25.2%	48.8%	58.9%	38.6%
Residual Std. Error	77.17	41.30	27.85	24.18	50.39	18.54	33.60	28.84	23.41	49.91	44.55

*Note*. Sample size = 500 scholars per country and 5,000 for the pooled sample. Cell entries of citations are final-entry standardized beta (*b*) coefficients. Coefficients effects accounted for robust standard errors based on bootstrapping to 1,000 resamples with biased corrected confidence set at 95% to assess statistical significance. Bootstrapped standard errors in brackets.

For the top 100 (H3a_2_), in most countries there is no statistically significant association between gender and citations after controlling for research productivity and h-index, as reported in Table 7. Only in the United States (*b =* .23, *p* < .001, ΔR^2^ = 5.5%) and in the pooled sample (*b =* .06, *p* < .05, ΔR^2^ = 0.4%), was there a statistically significant association, and contrary to our expectations, the association was positive, meaning that female scholars are statistically significant more cited than males. Accordingly, H3a_2_ was not empirically supported.

**Table 7:** Bootstrapped OLS regression predicting citations for the top 100 most productive scholars across the most productive countries in communication.

	(Top 100) Citations										
	United States	United Kingdom	China	Spain	Germany	India	Australia	Canada	Italy	Netherlands	TOTAL
Block 1											
Research Productivity	.48***	.47**	.15*	.48*	.39***	.00	.49**	.40	.65***	.73***	.56***
	(4.26)	(3.45)	(0.66)	(2.07)	(2.68)	(0.89)	(2.90)	(3.35)	(1.76)	(1.90)	(0.42)
ΔR^2^	28.1%	25.4%	2.5%	31.6%	17.3%	0%	26.9%	16.6%	51.1%	58.1%	35.6%
Block 2											
H-index	.28**	.20*	.03	.28**	.08	-.05	.24**	.01	.19	.10	.17***
	(1.01)	(0.41)	(0.20)	(0.99)	(0.69)	(0.13)	(0.53)	(0.23)	(0.58)	(0.68)	(0.18)
ΔR^2^	5.5%	0.5%	0.1%	6.6%	0.6%	0.3%	6%	0%	2.9%	0.9%	2.6%
Variable of Interest											
Gender_(female)_	.23***	-.09	.10	.06	-.00	.00	-.04	-.02	.03	.01	.06*
	(20.06)	(12.94)	(7.31)	(7.18)	(12.95)	(8.96)	(8.59)	(11.81)	(11.32)	(17.84)	(4.57)
ΔR^2^	5.5%	0.9%	1%	0.3%	0%	0%	0.2%	0%	0.1%	0%	0.4%
R^2^	39.2%	31.7%	3.5%	38.6%	17.9%	0.3%	33.2%	16.7%	54.1%	59%	38.5%
Adj.R^2^	37.3%	29.5%	0.5%	36.7%	15.4%	0%	31.1%	14.1%	52.7%	57.7%	38.3%
Residual Std. Error	105.62	64.42	29.45	39.71	63.50	25.71	46.94	57.32	43.52	83.51	70.57

*Note*. Sample size = 100 scholars per country and 1,000 for the pooled sample. Cell entries of citations are final-entry standardized beta (*b*) coefficients. Coefficients effects accounted for robust standard errors based on bootstrapping to 1,000 resamples with biased corrected confidence set at 95% to assess statistical significance. Bootstrapped standard errors in brackets.

Testing gender differences in h-index for the top 500 scholars (H3b_1_), results of the bootstrapped OLS-regression analysis in Table 8 revealed that in all countries under examination and the pooled sample, there was a statistically significant and negative association between gender and h-index, meaning that female scholars have less h-index than male scholars after controlling for research productivity and citation levels. All standardized beta values ranged between -.10 (China) and -.20 (Italy), while the explanatory power of gender in explaining h-index ranged between 12.1% (the United Kingdom) and 2.2% (China). Accordingly, H3b_1_ was fully supported.

**Table 8:** Bootstrapped OLS regression predicting h-index for the top 500 most productive scholars across the most productive countries in communication.

	(Top 500) h-index										
	United States	United Kingdom	China	Spain	Germany	India	Australia	Canada	Italy	Netherlands	TOTAL
Block 1											
Research Productivity	.03	.07	.06	-.10	.01	.10*	-.17***	.07	.00	.09	.00
	(0.14)	(0.36)	(0.20)	(0.15)	(0.22)	(0.19)	(0.25)	(0.24)	(0.16)	(0.22)	(0.06)
ΔR^2^	2.9%	4.6%	0.7%	1.7%	2.2%	1.6%	0.5%	1.5%	3.7%	6%	2.1%
Block 2											
Citations	.24***	.23**	.05	.35***	.26***	.10*	.19**	.09	.26***	.19**	.23***
	(0.00)	(0.36)	(0.01)	(0.01)	(0.01)	(0.02)	(0.01)	(0.02)	(0.02)	(0.00)	(0.00)
ΔR^2^	4%	4.4%	0.3%	7.5%	5.6%	1.2%	3%	0.6%	4.2%	1.7%	3.4%
Variable of Interest											
Gender_(female)_	-.11**	-.17***	-.10*	-.15***	-.16***	-.16**	-.12**	-.12**	-.20***	-.17***	-.14***
	(0.98)	(0.02)	(0.98)	(0.52)	(0.94)	(0.70)	(1.12)	(1.13)	(0.87)	(0.90)	(0.29)
ΔR^2^	1.4%	3.1%	1.2%	2.3%	2.6%	2.6%	1.6%	1.5%	4%	3%	1.9%
R^2^	8.3%	12.1%	2.2%	11.4%	10.4%	5.4%	5%	3.6%	12%	10.7%	7.5%
Adj.R^2^	7.8%	11.6%	1.6%	10.9%	9.8%	4.8%	4.5%	3%	11.4%	10.1%	7.5%
Residual Std. Error	10.91	12.28	11.63	6.25	10.81	8.95	12.80	13.46	10.44	10.40	11.22

*Note*. Sample size = 500 scholars per country and 5,000 for the pooled sample. Cell entries of citations are final-entry standardized beta (*b*) coefficients. Coefficients effects accounted for robust standard errors based on bootstrapping to 1,000 resamples with biased corrected confidence set at 95% to assess statistical significance. Bootstrapped standard errors in brackets.

Finally, testing gender differences in h-index for the top 100 scholars (H3b_2_), results of the bootstrapped OLS-regression analysis in Table 9 revealed a statistically significant and negative association between gender and h-index in the United States (*b =* -.23, *p* < .05, ΔR^2^ = 5.2%), the United Kingdom (*b =* -.22, *p* < .05, ΔR^2^ = 4.7%), Spain (*b =* -.20, *p* < .05, ΔR^2^ = 4.2%), Germany (*b =* -.24, *p* < .01, ΔR^2^ = 5.8%), Italy (*b =* -.24, *p* < .05, ΔR^2^ = 5.7%), and the pooled sample (*b =* -.13, *p* < .001, ΔR^2^ = 1.7%). For China, India, Australia, Canada, and the Netherlands, the association was not statistically significant. Accordingly, H3b_2_ was empirically supported in 6 out of 11 predictions.

**Table 9:** Bootstrapped OLS regression predicting h-index for the top 100 most productive scholars across the most productive countries in communication.

	(Top 100) h-index										
	United States	United Kingdom	China	Spain	Germany	India	Australia	Canada	Italy	Netherlands	TOTAL
Block 1											
Research Productivity	-.00	-.06	.16	.08	.22*	.05	-.08	.17*	.11	.15	.04
	(0.25)	(0.60)	(0.34)	(0.19)	(0.37)	(0.31)	(0.44)	(0.29)	(0.23)	(0.39)	(0.10)
ΔR^2^	4.4%	0.9%	3%	10.1%	8.4%	0.2%	0.8%	3.3%	12.2%	11.4%	3.9%
Block 2											
Citations	.38**	.26*	.03	.35*	.08	-.05	.33*	.01	.31*	.21	.25***
	(0.01)	(0.02)	(0.03)	(0.01)	(0.01)	(0.03)	(0.02)	(0.02)	(0.02)	(0.01)	(0.00)
ΔR^2^	7.3%	7.1%	0.1%	8.7%	0.7%	0.3%	8.2%	0%	5.2%	1.9%	3.9%
Variable of Interest											
Gender_(female)_	-.23*	-.22*	-.05	-.20*	-.24**	-.11	.03	-.08	-.24**	-.09	-.13***
	(2.03)	(2.93)	(2.18)	(0.80)	(1.74)	(2.23)	(1.97)	(2.99)	(2.09)	(2.44)	(0.73)
ΔR^2^	5.2%	4.7%	0.3%	4.2%	5.8%	1.3%	0.1%	0.8%	5.7%	0.8%	1.7%
R^2^	16.9%	12.7%	3.4%	23%	14.9%	1.8%	9.1%	4.1%	23.1%	14%	9.5%
Adj.R^2^	14.3%	10%	0.3%	20.6%	12.2%	0%	6.2%	1.1%	20.7%	11.3%	9.2%
Residual Std. Error	10.10	16.85	12.38	4.73	8.75	13.85	9.66	14.45	11.02	11.20	11.98

*Note*. Sample size = 100 scholars per country and 1,000 for the pooled sample. Cell entries of citations are final-entry standardized beta (*b*) coefficients. Coefficients effects accounted for robust standard errors based on bootstrapping to 1,000 resamples with biased corrected confidence set at 95% to assess statistical significance. Bootstrapped standard errors in brackets.

## Discussion

Analyzing the representation and impact of female researchers is a necessary task for uncovering the—often unequal—configuration of disciplines in terms of gender. Structural inequalities among epistemic communities, regardless of their nature, are a detriment to diverse and plural scientific and academic spaces. Often, diversity among those who produce knowledge is linked with the diversity of the knowledge that is being produced. Therefore, epistemic spaces that are more homogeneous in terms of the social identity of their members tend to produce knowledge that is less equipped to address the complexity and plurality of the world. As stated before, this phenomenon is referred to as hermeneutical injustice [17], resulting in a lack of epistemic tools to address the lived experience of those who lack epistemic authority and voice.

Success in academic spaces, which are considered a “credibility economy” [17], is the result of being heard and recognized as an authority by others, thus having impact on one’s own epistemic community. Scientific impact, gauged by metrics like citations and the h-index, is directly associated with a researcher’s symbolic authority, increasing with greater impact. However, we know that science capital is not only dependent on epistemic qualities, but also on the social identity of individuals [36], as social meanings permeate within academia. In particular, as we have priorly stated, gender meanings have been shown to constantly permeate academic environments, which unchain into a systematic loss of trust and authority suffered by women researchers referred to as the Matilda effect [19]. This phenomenon, together with many other forms of social and epistemic injustice suffered by female researchers [16], undermines women’s academic careers in the long run, resulting in a consistent, and systematic loss of female talent throughout the stages of the academic career: the leaky-pipeline phenomenon [44].

These dynamics are also true for the field of communication research, where, as priorly shown, female scholars continue to occupy a secondary role, being systematically less cited [6–8, 27], less considered as referents [28], and ultimately less able to succeed in academia in the long-term [47]. Addressing current gender differences in representation and impact in the field of communication is a first step towards identifying how female scholars are affected by current scientific dynamics of authority, as well as towards counteracting such dynamics.

Regarding gender representation, our results first show that, assuming an equal (50/50) gender representation among the top 500 scholars of the most productive countries in communication research (H1a), there are less women than men in almost all countries under examination. Despite the widespread gender inequality that affects every country studied, we find that United States, Spain, the Netherlands, and Australia are the countries where gender differences in representation among the top 500 scholars are less pronounced. These countries are closer to equality, with some of them having a generally greater number of female scholars. Examining gender representation among the top 100 scholars (H1b), we find a more equal representation, with China, India, and Germany still constituting the most unequal geographies. Still, only the Netherlands has more women than men among the top 100 scholars, which proves the strength of female scientific production in one of the most important and most impactful countries in communication research worldwide.

To answer RQ1, we studied gender proportions assuming the proportions of the pooled sample, for both the top 500 (RQ1a) and the top 100 (RQ1b). In most of the countries, the gender differences diminished, and compared to the pooled sample, there are statistically significant more females in Spain, the United States, and the Netherlands, while statistically significant less women in India (in this case, also for the top 100, RQ1b) and Germany among the top 500 (RQ1a). While the first one is a developing nation, a space where gender disparities can often be more significant [49: 85], Germany is considered a developed country, as well as a member of the European Union, where gender equality policies—including those linked to media and communication [50]—have been in place for decades. In cases like this, further steps must be taken to ensure that gender representation is as equal as possible.

This widespread unequal distribution of male and female researchers among the top 500 scholars of the most productive countries in communication research may not be coincidental. Rather, it may be the result of gendered structural factors such as those mentioned priorly in this paper. Those structural factors may affect female scholars because of their gender identity, causing harm to their careers and their impact, and resulting in unequally distributed disciplines where, despite steps towards plurality, women still occupy the lower, less impactful, and less productive steps of the academic career. Structural differences affecting female scholars must be better understood in order to be recognized and corrected, which is the goal of our paper.

Regarding impact (H2), when analyzing citations (not controlling for productivity and h-index; see Table 5) in the 2019–2022 period, we find that none of the studied countries show significant differences between male and female scholars among the top 500 (H2a_1_). However, when considering a global picture of all of the countries, we see that women are statistically more cited than men, which refutes our starting hypothesis. Despite being less represented among the most productive scholars, women and their contributions have a higher impact. We could argue that women need a lesser volume of representation to have a greater impact.

Among the top 100 scholars (H2a_2_), only India and the United Kingdom show statistically significant gender differences regarding citations, with women being less cited than men in the 2019–2022 period. Women are also less cited than men in the pooled sample. However, the fact that female scholars receive more citations than men in the rest of the countries studied shows an encouraging picture of the situation of women in the field of communication, a picture that contradicts the Matilda effect and the structural and historic lack of authority suffered by women researchers.

When studying cumulative indicators, like the h-index (without controlling for productivity and citations), we see the impact of cumulative differences and structural inequalities. Among the top 500 scholars (H2b_1_) in every country (including the pooled sample), except for the United States and Canada, women have a significantly lesser h-index than their male colleagues. This, together with the higher rate of citations received by female scholars when looking at shorter or still periods of time, suggests that most countries still display cumulative disadvantages affecting women academics. The United States and Canada appear to be the two countries where cumulative differences affecting female scholars in communication are not statistically significant. This might be due to a longer tradition of stable female careers in communication in both countries, where the first women to “jump the fence” [51] and gain full professorship positions did so already in the 1960s and 1970s, thus breaking glass ceilings sooner than in other geographical locations.

Among the top 100 scholars (H2b_2_), the situation regarding h-index differences is more balanced. Only in three countries (the United Kingdom, Germany, and Italy) and the pooled sample are gender differences statistically significant, with female scholars having less h-index than males. When we control for productivity and h-index, however, we find that, regarding citations (see Table 6) among the top 500 scholars (H3a_1_), in most countries gender differences in citations are not statistically significant. In the countries where those differences do exist, surprisingly, the results refute our hypothesis (H3). With the same level of productivity and h-index, for example, women researchers are more cited than men in the studied period (2019–2022) both in Spain and in the pooled sample. This proves again that, in shorter periods of time, women’s contributions are generally recognized by their peers and gain research traction.

Regarding the top 100 scholars (H3a_2_), when controlling for productivity and h-index, the pattern is similar to that shown before. In most countries, we find no statistically significant gender differences in citations. Only the United States and the pooled sample have differences, and again, contrary to our hypothesis (H3a_2_), women are more cited than men in the analyzed period (2019–2022).

Thus, our most relevant findings are those related to the h-index (H3b). We showed the existence of cumulative gender differences—those structural differences that affect academic careers in longer periods of time—even controlling for researchers’ productivity and citation scores. Regarding the top 500 scholars (H3b_1_), women have a statistically significant lesser h-index in every country and in the pooled sample with the same level of productivity and citations than men. Among the top 100 scholars, when controlling for productivity and citations, women have a lesser h-index in the United States, the United Kingdom, Spain, Germany, Italy, and the pooled sample, but not in the rest of the countries.

## Conclusions

In conclusion, we found that women are still not well-represented among the most productive scholars in communication research. This inequality in representation is the result of long-standing epistemic and social traditions that undermine female scholars’ careers and women’s authority in science. The impact of women in the short-run is similar and even higher than men’s in most countries. This might be the result of gender politics in place [44], a higher number of younger female scholars and women in the lower stages of the academic career [45], and a growing conscience about the need for gender equality [44]. With a changing environment in terms of gender parity, women become active and recognized members of their academic and epistemic communities. However, cumulative differences are still decisively impacting female careers in the field of communication in the long-run. Women have more trouble stabilizing their careers—thus, the leaky pipeline phenomenon [44]—and ultimately have more challenges becoming referents, or finding a place among the field’s elite scholars. Cumulative differences are often rooted in long-standing epistemic beliefs [11] that are hard to overcome, in the impact of traditional social meanings and demands that complicate the success of women in a high-demanding space like academia, and even in widespread meanings about scientists and authority figures [15, 17]. All in all, pluralizing our epistemic communities in terms of gender is necessary for producing more complex and more diverse knowledge. However, there is still work to be done in order to achieve actual gender parity in the field of communication research. Recognizing the lost, historical contributions of women to the field, as well as promoting a tradition of female work in communication can help us produce more plural forms of knowledge. Ultimately, it will allow us to overcome the stereotypes and long-standing prejudices against women researchers that favor cumulative harm toward them and their careers.

## Supporting information

S1 Table Bootstrapped OLS regression predicting citations for the top 500 most productive scholars across the most productive countries in communication. (DOCX)

S2 Table Bootstrapped OLS regression predicting citations for the top 100 most productive scholars across the most productive countries in communication. (DOCX)

S3 Table Bootstrapped OLS regression predicting h-index for the top 500 most productive scholars across the most productive countries in communication. (DOCX)

S4 Table Bootstrapped OLS regression predicting h-index for the top 100 most productive scholars across the most productive countries in communication. (DOCX)

S1 Data (XLSX)

## References

[1] Cole J. & Zukerman H. (1984). The productivity puzzle. *Advances in Motivation and Achievement*, 2, 17–256.

[2] Fox M. F. (2020). Gender, science, and academic rank: Key issues and approaches. *Quantitative Science Studies*, 1(3), 1001–1006. 10.1162/qss_a_00057

[3] Astegiano J., Sebastián-González E., & de Toledo C. (2019). Unraveling the gender productivity gap in science: A meta-analytical review. *Royal Society Open Science*, 6(6), 181566. 10.1098/rsos.181566 31312468 PMC6599789

[4] Chan H. & Torgler B. (2020). Gender differences in performance of top cited scientists by field and country. *Scientometrics*, 125(3), 2421–2447. 10.1007/s11192-020-03733-w

[5] Larivière V., Ni C., Gingras Y., Cronin B., & Sugimoto C. (2013). Bibliometrics: Global gender disparities in science. *Nature*, 504(7479), 211–213. doi: 10.1038/504211a 24350369

[6] Knobloch-Westerwick S. & Glynn C. (2013). The Matilda effect—Role congruity effects on scholarly communication: A citation analysis of Communication Research and Journal of Communication articles. *Communication Research*, 40(1), 3–26. 10.1177/0093650211418339

[7] Knobloch-Westerwick S., Glynn C., & Huge M. (2013). The Matilda effect in science communication: An experiment on gender bias in publication quality perceptions and collaboration interest. *Science Communication*, 35(5), 603–625. 10.1177/1075547012472684

[8] Rajkó A., Herendy C., Goyanes M., & Demeter M. (2023). The Matilda effect in communication research: The effects of gender and geography on usage and citations across 11 countries. *Communication Research*, 0(0). 10.1177/00936502221124389

[9] Segado-Boj F., Prieto-Gutiérrez J.J., & Quevedo-Redondo R. (2021). El efecto Matilda en la red de coautorías hispanoamericana en comunicación. *Revista Mediterránea de Comunicación*, 12(2), 77–95.

[10] DiPrete T. A. & Eirich G. M. (2006). Cumulative advantage as a mechanism for inequality: A review of theoretical and empirical developments. *Annual Review of Sociology*, 32, 271–297. 10.1146/annurev.soc.32.061604.123127

[11] Lloyd G. (2015). The man of reason. In Garry A & Pearshall M (Eds.), *Women*, *knowledge*, *and reality* (pp. 149–165). Routledge.

[12] Carli L., Alawa L., Lee Y., Zhao B., & Kim E. (2016). Stereotypes about gender and science: Women ≠ scientists. *Psychology of Women Quarterly*, 40(2), 244–260. 10.1177/0361684315622645

[13] Cheng S. (2020). *Careers versus children*: *How childcare affects the academic tenure-track gender gap* [Job market paper, Harvard University]. Harvard Scholar Papers. https://scholar.harvard.edu/files/sdcheng/files/sdcheng_kids_jmpv6.pdf

[14] Dolamore S., Henderson A., & Carrizales T. (2021). Structural obstacles for women in academia. *Journal of Public Management & Social Policy*, 28(1), 9. https://digitalscholarship.tsu.edu/jpmsp/vol28/iss1/9

[15] Eagly A. H. & Karau S. J. (2002). Role congruity theory of prejudice toward female leaders. *Psychological Review*, 109(3), 573. doi: 10.1037/0033-295x.109.3.573 12088246

[16] Shorey S., Chua C., & Yap Seng C. (2023). Turbulent academic journey of female academics: A meta-synthesis. *International Journal of Inclusive Education*, 1–21. 10.1080/13603116.2023.2227862

[17] Fricker M. (2007). *Epistemic injustice*: *Power and the ethics of knowing*. Oxford University Press.

[18] Ceci S. J., Kahn S., & Williams W. (2023). Exploring gender bias in six key domains of academic science: An adversarial collaboration. *Psychological Science in the Public Interest*, 24(1), 15–73. doi: 10.1177/15291006231163179 37098793 PMC10394402

[19] Rossiter M. (1993). The Matthew/Matilda effect in science. *Social Studies of Science*, 23(2), 325–341. https://www.jstor.org/stable/285482

[20] Anderson K., Feldner S., McDorman K., Pierce J., Procopio C., Sheeler K., et al. (2004). Voices about choices: The role of female networks in affirming life choices in the academy. *Women’s Studies in Communication*, 27(1), 88–110. 10.1080/07491409.2004.10162467

[21] Bagilhole B. (2017). Being different is a very difficult row to hoe: Survival strategies of women academics. In Quinn J, Davies S, & Lubelska C (Eds.), *Changing the subject* (pp. 15–28). Taylor & Francis.

[22] Teich E., Kim J., Lynn C., Simon S., Klishin A., Szymula K., et al. (2022). Citation inequity and gendered citation practices in contemporary physics. *Nature Physics*, 18(10), 1161–1170. 10.1038/s41567-022-01770-1

[23] Gasser C. & Shaffer K. (2014). Career development of women in academia: Traversing the leaky pipeline. *The Professional Counselor*, 4(4), 332–352. http://hdl.handle.net/11603/5410

[24] Monroe K., Choi J., Howell E., Lampros-Monroe C., Trejo C., & Perez V. (2014). Gender equality in the ivory tower, and how best to achieve it. *PS*: *Political Science & Politics*, 47(2), 418–426.

[25] Colgan J. (2017). Gender bias in international relations graduate education? New evidence from syllabi. *PS*: *Political Science & Politics*, 50(2), 456–460. 10.1017/S1049096516002997

[26] Ginther D. & Kahn S. (2009). Does science promote women? Evidence from academia 1973–2001. In Freeman R & Goroff D (Eds.), *Science and engineering careers in the United States*: *An analysis of markets and employment* (pp. 163–194). UCP.

[27] Wang X., Dworkin J., Zhou D., Stiso J., Falk E., Bassett D., et al. (2021). Gendered citation practices in the field of communication. *Annals of the International Communication Association*, 45(2), 134–153, doi: 10.1080/23808985.2021.1960180 34541322 PMC8443000

[28] García-Jiménez L., Torrado-Morales S., & Díaz J. (2022). El rol de la mujer en la ciencia y la docencia en comunicación: Análisis a partir de los programas universitarios en España. *Revista de Comunicación*, 21(2), 91–113. 10.26441/RC21.2-2022-A5

[29] Pooley J. & Socolow M. (2013). Checking up on *The Invasion from Mars*: Hadley Cantril, Paul Lazarsfeld, and the making of a misremembered classic. *International Journal of Communication*, 7, 29. https://doi.org/1932–8036/20130005

[30] Dorsten A. M. (2012). “Thinking dirty”: Digging up three founding “matriarchs” of communication Studies. *Communication Theory*, 22(1), 25–47. 10.1111/j.1468-2885.2011.01398.x

[31] Rowland A. & Simonson P. (2014). The founding mothers of communication research: Toward a history of a gendered assemblage. *Critical Studies in Media Communication*, 31(1), 3–26. 10.1080/15295036.2013.849355

[32] Treichler P. & Wartella E. (2022). Interventions: Feminist theory and communication studies. In Wartella E & Treichler P (Eds.), *Feminist critiques of popular culture* (pp. 1–18). Routledge. 10.4324/9781315074955

[33] García-Jiménez L. & Herrero E. (2022). Including female voices in the stories we tell about communication research: Memories and narratives of women in academia. *Communication Theory*, 32(2), 289–297. 10.1093/ct/qtac002

[34] McCusker D. (2019). What is the harm in gendered citation practices? *Philosophy of Science*, 86(5), 1041–1051. 10.1086/705495

[35] Buela-Casal G. (2010). Scientific journal impact indexes and indicators for measuring researchers’ performance. *Revista de psicodidáctica*, 15(1), 3–19.

[36] Bourdieu P. (2004). *Science of science and reflexivity*. UCP.

[37] Cole J. (2000). A short history of the use of citations as a measure of the impact of scientific and scholarly work. In Cronin B & Barsky H (Eds.), *The web of knowledge*: *A festschrift in honor of Eugene Garfield* (pp. 281–300). ASIS.

[38] Hirsch J. & Buela-Casal G. (2014). The meaning of the h-index. *International Journal of Clinical and Health Psychology*, 14(2), 161–164. 10.1016/S1697-2600(14)70050-X

[39] Ali M. (2021). Forewarned is forearmed: the h-index as a scientometric. *Seminars in Ophthalmology*, 36, (1–2), 1–1). doi: 10.1080/08820538.2021.1894889 33734008

[40] Moore K., Cid-Martinez I., Toney J., Smith J., Kalb A., Shin J., et al. (2018). Who climbs the academic ladder? Race and gender stratification in a world of whiteness. *The Review of Black political economy*, 45(3), 216–244. 10.1177/0034644618813667

[41] Sá C., Cowley S., Martinez M., Kachynska N., & Sabzalieva E. (2020). Gender gaps in research productivity and recognition among elite scientists in the U.S., Canada, and South Africa. *PlOS ONE*, 15(10), e0240903. doi: 10.1371/journal.pone.0240903 33119671 PMC7595278

[42] Schiebinger L. (1991). *The mind has no sex*? *Women in the origins of modern science*. Harvard University Press. 10.1126/science.246.4936.1502 17756008

[43] Pell A. N. (1996). Fixing the leaky pipeline: Women scientists in academia. *Journal of Animal Science*, 74(11), 2843–2848. doi: 10.2527/1996.74112843x 8923199

[44] UNESCO. (2021). *UNESCO science report*: *The race against time for smarter development*. United Nations Educational.

[45] Gonzales L., Allum J., & Sowell R. (2013). *Graduate enrollment and degrees*: *2002 to 2012*. Council of Graduate Schools.

[46] Alter K. J., Clipperton J., Schraudenbach E., & Rozier L. (2020). Gender and status in American political science: Who determines whether a scholar is noteworthy? *Perspectives on Politics*, 18(4), 1048–1067. 10.1017/S1537592719004985

[47] Diezmann C. & Grieshaber S. (2019). *Women professors*: *Who makes it and how*? Springer.

[48] Sebo P. (2021). Performance of gender detection tools: A comparative study of name-to-gender inference services. *Journal of the Medical Library Association*, 109(3), 414–421. doi: 10.5195/jmla.2021.1185 34629970 PMC8485937

[49] Huyer S. (2015). Is the gender gap narrowing in science and engineering. In Schneegans S (Ed.), *UNESCO science report*: *Towards*, *2030* (pp. 85–103). United Nations Educational.

[50] Guerrero Cantarell R., Flury C., & Geiss M. (2023). From victims to economic assets. *History of Media Studies*, 3, 1–29. 10.32376/d895a0ea.6e09b010

[51] Dorsten A. M. (2016). Women in communication. *International Encyclopedia of Communication Theory and Philosophy*. John Wiley & Sons. 10.1002/9781118766804.wbiect106

