# Supplementary material for: Gender differences in representation, citations, and h-index: An empirical examination of the field of communication across the ten most productive countries
Source: PLoS One. 2024 Nov 20;19(11):e0312731. doi: 10.1371/journal.pone.0312731 (PMC11578513; doi:10.1371/journal.pone.0312731)
Supplement: S1 Table — (DOCX) [file pone.0312731.s001.docx]

**Table A1.** *Bootstrapped OLS regression predicting citations for the top 500 most productive scholars across the most productive countries in communication*

|  | (Top 500) Citations | | | | | | | | | | |  |
| --- | --- | --- | --- | --- | --- | --- | --- | --- | --- | --- | --- | --- |
|  | United States | United Kingdom | China | Spain | Germany | India | Australia | Canada | Italy | Netherlands | TOTAL | |
| Block 1 |  |  |  |  |  |  |  |  |  |  |  | |
| Research Productivity | .54***  (1.68) | .55***  (1.65) | .16***  (0.51) | .61***  (0.94) | .47***  (1.11) | .11  (0.72) | .52***  (1.25) | .50**  (2.27) | .68***  (1.35) | .76***  (1.05) | .60***  (0.44) | |
| ∆R^2^ | 30.1% | 31.5% | 2.6% | 37.7% | 22.9% | 1.4% | 27.7% | 25.1% | 46.8% | .58% | 36.4% | |
| Variable of Interest |  |  |  |  |  |  |  |  |  |  |  | |
| Gender_(female)_ | .03  (6.92) | -.07*  (3.64) | .02  (2.54) | .03  (2.09) | -.01  (4.45) | -.02  (2.00) | -.05  (2.96) | .02  (2.61) | -.03  (2.17) | -.01  (4.26) | .00  (1.24) | |
| ∆R^2^ | 0.1% | 0.6% | 0.1% | 0.1% | 0% | 0% | 0.3% | 0.1% | 0.1% | 0% | 0% | |
| R^2^ | 30.2% | 32% | 2.6% | 37.9% | 22.9% | 1.5 | 28% | 25.1% | 46.9% | 58.4% | 36.4% | |
| Adj.R^2^ | 29.9% | 31.7% | 2.3% | 37.6% | 22.6% | 1.1 | 27.7% | 24.8% | 46.7% | 58.2% | 36.3% | |
| Residual Std. Error | 78.84 | 42.12 | 27.87 | 25.20 | 51.86 | 18.64 | 34.04 | 28.91 | 23.89 | 50.31 | 45.37 | |

*Note.* Sample size = 500 scholars per country and 5,000 for the pooled sample. Cell entries of citations are final-entry standardized beta (*b*) coefficients. Coefficients effects accounted for robust standard errors based on bootstrapping to 1,000 resamples with biased corrected confidence set at 95% to assess statistical significance. Bootstrapped standard errors in brackets.
