# Supplementary material for: Gender differences in representation, citations, and h-index: An empirical examination of the field of communication across the ten most productive countries
Source: PLoS One. 2024 Nov 20;19(11):e0312731. doi: 10.1371/journal.pone.0312731 (PMC11578513; doi:10.1371/journal.pone.0312731)
Supplement: S2 Table — (DOCX) [file pone.0312731.s002.docx]

**Table A2.** *Bootstrapped OLS regression predicting citations for the top 100 most productive scholars across the most productive countries in communication*

|  | (Top 100) Citations | | | | | | | | | | |  |
| --- | --- | --- | --- | --- | --- | --- | --- | --- | --- | --- | --- | --- |
|  | United States | United Kingdom | China | Spain | Germany | India | Australia | Canada | Italy | Netherlands | TOTAL | |
| Block 1 |  |  |  |  |  |  |  |  |  |  |  | |
| Research Productivity | .54***  (4.32) | .48**  (3.49) | .15*  (0.62) | .56**  (2.10) | .41**  (2.74) | .00  (0.90) | .51**  (3.22) | .40  (3.60) | .71***  (2.08) | .76***  (1.75) | .59***  (0.83) | |
| ∆R^2^ | 28.1% | 25.4% | 2.5% | 31.6% | 17.3% | 0% | 26.9% | 16.6% | 51.1% | 58.1% | 35.6% | |
| Variable of Interest |  |  |  |  |  | 0% |  |  |  |  |  | |
| Gender_(female)_ | .19*  (22.55) | -.15  (12.98) | .09  (7.09) | .00  (7.34) | -.02  (13.07) | .00  (9.29) | -.04  (8.98) | -.02  (11.67) | -01  (9.86) | .00  (18.42) | .04  (4.61) | |
| ∆R^2^ | 3.7% | 2.2% | 1% | 0% | 0.1% | 0% | 0.2% | 0% | 0% | 0% | 0.2% | |
| R^2^ | 31.8% | 27.7% | 3.4% | 31.6% | 17.4 | 0% | 27.1% | 16.7% | 51.2% | 58.1% | 35.7% | |
| Adj.R^2^ | 30.4% | 26.2% | 1.4% | 30.2% | 15.7% | 0% | 25.6% | 15% | 50.2% | 57.2% | 35.6% | |
| Residual Std. Error | 111.21 | 65.92 | 29.32 | 41.69 | 63.39 | 25.61 | 48.77 | 57.03 | 44.66 | 83.99 | 72.12 | |

*Note.* Sample size = 100 scholars per country and 1,000 for the pooled sample. Cell entries of citations are final-entry standardized beta (*b*) coefficients. Coefficients effects accounted for robust standard errors based on bootstrapping to 1,000 resamples with biased corrected confidence set at 95% to assess statistical significance. Bootstrapped standard errors in brackets.
