# Supplementary material for: Gender differences in representation, citations, and h-index: An empirical examination of the field of communication across the ten most productive countries
Source: PLoS One. 2024 Nov 20;19(11):e0312731. doi: 10.1371/journal.pone.0312731 (PMC11578513; doi:10.1371/journal.pone.0312731)
Supplement: S3 Table — (DOCX) [file pone.0312731.s003.docx]

**Table A3.** *Bootstrapped OLS regression predicting h-index for the top 500 most productive scholars across the most productive countries in communication*

|  | (Top 500) h-index | | | | | | | | | | |
| --- | --- | --- | --- | --- | --- | --- | --- | --- | --- | --- | --- |
|  | United States | United Kingdom | China | Spain | Germany | India | Australia | Canada | Italy | Netherlands | TOTAL |
| Block 1 |  |  |  |  |  |  |  |  |  |  |  |
| Research Productivity | .16***  (0.11) | .20**  (0.34) | .07  (0.20) | .11**  (0.08) | .14**  (0.16) | .11**  (0.19) | -.06  (0.22) | .12***  (0.19) | .18**  (0.24) | .24***  (0.16) | .14***  (0.04) |
| ∆R^2^ | 2.9% | 4.6% | 0.7% | 1.7% | 2.2% | 1.6% | 0.5% | 1.5% | 3.7% | 6% | 2.1% |
| Variable of Interest |  |  |  |  |  |  |  |  |  |  |  |
| Gender_(female)_ | -.11**  (1.00) | -.19***  (1.02) | -.10**  (0.98) | -.13**  (0.57) | -.16***  (0.91) | -.16***  (0.69) | -.113**  (1.17) | -.12**  (1.15) | -.21***  (0.92) | -.17***  (0.89) | -.13***  (0.31) |
| ∆R^2^ | 1.2% | 3.8% | 1.2% | 1.9% | 2.7% | 2.7% | 1.9% | 1.5% | 4.4% | 3.1% | 1.9% |
| R^2^ | 4.1% | 8.4% | 1.8% | 3.6% | 4.9% | 4.2% | 2.4% | 2.9% | 8.1% | 9.1% | 4.1% |
| Adj.R^2^ | 3.7% | 8% | 1.4% | 3.2% | 4.5% | 3.9% | 2% | 2.5% | 7.7% | 8.7% | 4% |
| Residual Std. Error | 11.15 | 12.52 | 11.63 | 6.51 | 11.12 | 9.00 | 12.97 | 13.49 | 10.66 | 10.48 | 11.43 |

*Note.* Sample size = 500 scholars per country and 5,000 for the pooled sample. Cell entries of citations are final-entry standardized beta (*b*) coefficients. Coefficients effects accounted for robust standard errors based on bootstrapping to 1,000 resamples with biased corrected confidence set at 95% to assess statistical significance. Bootstrapped standard errors in brackets.
