# Supplementary material for: Gender differences in representation, citations, and h-index: An empirical examination of the field of communication across the ten most productive countries
Source: PLoS One. 2024 Nov 20;19(11):e0312731. doi: 10.1371/journal.pone.0312731 (PMC11578513; doi:10.1371/journal.pone.0312731)
Supplement: S4 Table — (DOCX) [file pone.0312731.s004.docx]

**Table A4.** *Bootstrapped OLS regression predicting h-index for the top 100 most productive scholars across the most productive countries in communication*

|  | (Top 100) h-index | | | | | | | | | | |
| --- | --- | --- | --- | --- | --- | --- | --- | --- | --- | --- | --- |
|  | United States | United Kingdom | China | Spain | Germany | India | Australia | Canada | Italy | Netherlands | TOTAL |
| Block 1 |  |  |  |  |  |  |  |  |  |  |  |
| Research Productivity | .19*  (0.21) | .05  (0.58) | .17  (0.33) | .28**  (0.11) | .25**  (0.36) | .05  (0.31) | .08  (0.35) | .18*  (0.25) | .34*  (0.36) | .31*  (0.31) | .19***  (0.09) |
| ∆R^2^ | 4.4% | 0.9% | 3% | 10.1% | 8.4% | 0.2% | 0.8% | 3.3% | 12.2% | 11.4% | 3.9% |
| Variable of Interest |  |  |  |  |  |  |  |  |  |  |  |
| Gender_(female)_ | -.16  (2.13) | -.26*  (3.06) | -.05  (2.16) | -.20*  (0.87) | -.24**  (1.71) | -.11  (2.18) | .01  (2.07) | -.08  (2.96) | -.24**  (2.16) | -.08  (2.43) | -.12***  (0.72) |
| ∆R^2^ | 2.6% | 6.7% | 0.3% | 4.2% | 5.9% | 1.3% | 0% | 0.8% | 6% | 0.7% | 1.5% |
| R^2^ | 6.9% | 7.7% | 3.2% | 14.3% | 14.3% | 1.5% | 0.8% | 4.1% | 18.2% | 12.1% | 5.4% |
| Adj.R^2^ | 5% | 5.8% | 1.2% | 12.5% | 12.5% | 0% | 0% | 2.1% | 16.5% | 10.3% | 5.2% |
| Residual Std. Error | 10.63 | 17.24 | 12.32 | 4.97 | 8.74 | 13.79 | 10.04 | 14.37 | 11.31 | 11.26 | 12.25 |

*Note.* Sample size = 100 scholars per country and 1,000 for the pooled sample. Cell entries of citations are final-entry standardized beta (*b*) coefficients. Coefficients effects accounted for robust standard errors based on bootstrapping to 1,000 resamples with biased corrected confidence set at 95% to assess statistical significance. Bootstrapped standard errors in brackets.
